# Short-term postoperative outcomes of gastric adenocarcinoma patients treated with curative intent in low-volume centers

**DOI:** 10.1186/s12957-022-02804-x

**Published:** 2022-10-17

**Authors:** Francisco-Javier Lacueva, Javier Escrig-Sos, Roberto Marti-Obiol, Carmen Zaragoza, Fernando Mingol, Miguel Oviedo, Nuria Peris, Joaquin Civera, Amparo Roig, on behalf of the RECEG-CV group, Consol Sabater, Vicente Espert, Gonzalo Todoli, María-José Cases, Mario Mella, Fernando Lopez-Mozos, Silvia Carbonell, Marcos Bruna, Claudia Mulas, Ramon Trullenque, José-Antonio Barreras, Luis Gomez, Cristina Sancho, Javier Aguilo, Jose-Manuel Navarro, Antonio Compañ, Alicia Calero, Enrique Canelles, Erick Montilla, Rodolfo Rodriguez, Yannko Gonzalez, Alejandro Moya, Xavier Barber, Jose Puche, Francisco Asencio

**Affiliations:** 1 Hospital General Universitario de Elche, Elche, Spain; 2 Hospital General Universitario de Castellón, Castellón de la Plana, Spain; 3 Hospital Clínico Universitario de Valencia, Valencia, Spain; 4 Hospital General Universitario de Alicante, Alicante, Spain; 5 Hospital Universitario y Politécnico La Fe de Valencia, Valencia, Spain; 6 Hospital General Universitario de Valencia, Valencia, Spain; 7 Hospital Universitario Doctor Peset de Valencia, Valencia, Spain; 8 Hospital Universitario Arnau de Vilanova de Valencia, Valencia, Spain; 9 Hospital Lluis Alcanyis de Xativa, Valencia, Spain

**Keywords:** Gastric cancer, Gastrectomy, Postoperative outcomes, Postoperative mortality, Failure to rescue, Age

## Abstract

**Background:**

Quality standards in postoperative outcomes have not yet been defined for gastric cancer surgery. Also, the effect of centralization of gastric cancer surgery on the improvement of postoperative outcomes continues to be debated. Short-term postoperative outcomes in gastric carcinoma patients in centers with low-volume of annual gastrectomies were assessed. The effect of age on major postoperative morbidity and mortality was also analyzed.

**Methods:**

Patients with gastric or gastroesophageal junction Siewert III type carcinomas who underwent surgical treatment with curative intent between January 2013 and December 2016 were included. Data were obtained from the population-based surgical registry Esophagogastric Carcinoma Registry of the Comunitat Valenciana (RECEG-CV). The RECEG-CV gathers information on demographic characteristics and comorbidity, preoperative study and neoadjuvant treatment, surgical procedure, pathological study, postoperative outcomes, and follow-up. Seventeen hospitals belonging to the public network participated in this registry.

**Results:**

Data from 591 patients were analyzed. Postoperative major morbidity occurred in 154 (26.1%) patients. Overall 30-day or in-hospital mortality, and 90-day postoperative mortality rates were 8.6% and 10.1% respectively. Failure-to-rescue was 39% and it was significantly higher in patients aged 75 years or older in comparison with younger patients (55.3% vs 23.1% *p* < 0.001).

In the multivariable analysis, age ≥ 75 years (*p* = 0.029), laparoscopic approach (*p* = 0.005), and total gastrectomy (*p* = 0.005) were associated with major postoperative morbidity. Age ≥ 75 years (*p* = 0.027), pulmonary complications (*p* = 0.001), cardiac complications (*p* = 0.001), leakage (*p* = 0.003), and hemorrhage (*p* = 0.013) were associated with postoperative mortality.

**Conclusions:**

Centralization of gastric adenocarcinoma treatment in centers with higher annual caseload should be considered to improve the short-term postoperative outcomes in low-volume centers. Patients aged 75 or older had a significantly increased risk of major postoperative morbidity and mortality, and higher failure-to-rescue.

## Background

Incidence of gastric adenocarcinoma shows a progressive drop over the last decades in contrast with the increasing incidence of gastroesophageal junction (GEJ) adenocarcinomas in Western countries [1]. However, gastric adenocarcinoma is still the third leading cause of cancer-related death worldwide and long-term survival remains poor even in patients treated with curative intent [1, 2]. Surgery is the main treatment for locally advanced disease, usually combined with perioperative chemotherapy or adjuvant treatment [3, 4].

Likewise, postoperative morbidity and mortality after gastrectomy in patients with gastric and GEJ adenocarcinomas are even higher than those recorded after esophagectomy with noteworthy variations among Western countries [5, 6]. Standardization in reporting postoperative complications has been claimed to allow for comparison among different studies and for quality improvement [7].

There is a trend towards centralization of gastric cancer treatment in centers with a minimum of 20 gastrectomies annually to reduce morbidity and mortality rates, similarly to what has been done with the treatment of esophageal cancer [8–10]. However, the beneficial effect of centralization on postoperative outcomes is still controversial for gastric cancer surgery [11, 12].

Population-based cancer registries are useful for monitoring and improving clinical outcomes, permitting also comparison among centers or countries and for promoting epidemiological research [10–12]. Completeness, accuracy, and data verification are crucial to assure the quality of clinical registries [13, 14]. Control charts have been shown to be useful tools for monitoring and identifying deterioration of clinical outcomes and are increasingly used for quality control in surgical oncology [15].

The main aim of this study was to assess short-term postoperative outcomes in gastric adenocarcinoma patients surgically treated with curative intent in centers with low annual gastrectomies volume. The effect of age on major postoperative morbidity and mortality was also analyzed.

## Methods

### Patients

**Fig. 1:**
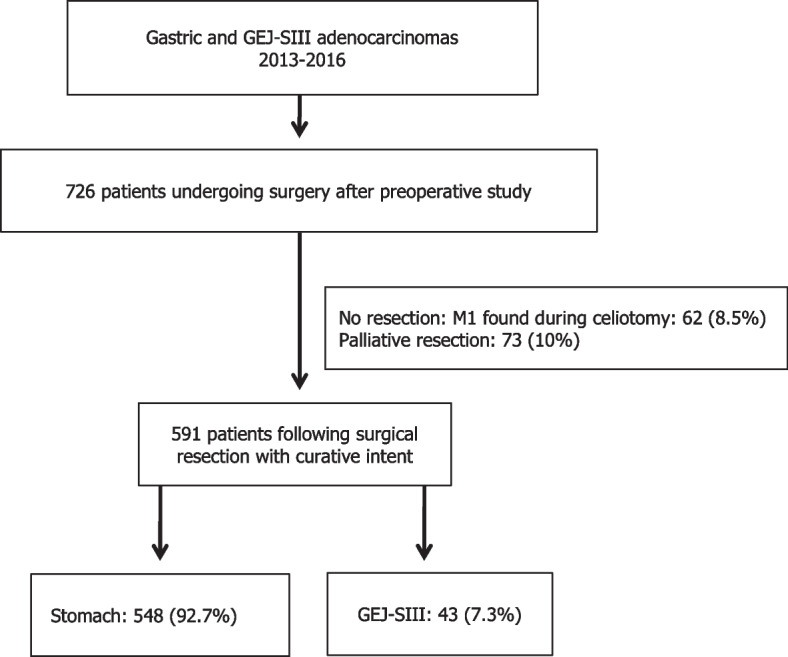
Flowchart of patients with gastric or gastroesophageal junction Siewert III type adenocarcinomas initially undergoing surgery and eventually following a surgical resection with curative intent. Legend: GEJ-SIII: Gastroesophageal junction Siewert III type

Data were obtained from the Esophagogastric Cancer Registry of the Comunitat Valenciana (RECEG-CV). The Comunitat Valenciana is an autonomous region in Eastern Spain with a population of more than 5 million inhabitants. All patients with gastric or GEJ Siewert III type (GEJ-SIII) adenocarcinomas treated with curative intent during a 4-year period (2013–2016) were included (Fig. 1). Seventeen of the 24 hospitals of the public network of the Comunitat Valenciana participated, even though 3 hospitals joined the RECEG-CV only for the last 2-year period (2015–2016). No center performed at least 20 gastrectomies annually, and for that reason all the hospitals were considered low-volume centers, although hospitals performing less than 10 gastrectomies per year were also considered as very low-volume hospitals.

### RECEG-CV registry

The RECEG-CV is a population-based surgical online registry included into the NEOS platform that belongs to the Cancer Information System of the Comunitat Valenciana. It was fostered by the Valencian Surgical Society and the Epidemiological Studies and Health Statistics Service of the Comunitat Valenciana. The RECEG-CV gathers only clinical data from esophageal and gastric carcinoma patients following surgical treatment. Hospitals belonging to the public network feed data to this registry on a voluntary basis since 2013. Only one or 2 surgeons from each hospital are authorized to introduce data from their center into this online registry but are not allowed to check data from other centers. Only two investigators (F-JL and JE) had access to the entire data set in order to check the completeness and reliability of the data. Whenever some data seemed implausible or erroneous one of the two investigators contacted the authorized surgeons in the corresponding hospital for verification.

The study was approved by the Ethics Committee of the General University Hospital of Elche (PI 46/2022).

### Data set

The RECEG-CV gathers information of 55 items allocated in 6 sections: demographic characteristics and comorbidity (6 items), preoperative study and neoadjuvant treatment (9 items), surgical procedure (9 items), pathological study (11 items), postoperative outcomes (11 items), and follow-up (8 items). This data set was designed as a reduced version of the online data set of the Spanish EURECCA Esophagogastric Cancer Registry (SEEGCR) [14].

Gastric and GJ-SIII tumors were staged according to the 7th edition of the UICC TNM classification [16] American Society of Anesthesiologists (ASA) score and Charlson index were collected in the comorbidity section. Pulmonary, cardiac, other medical complications, leakage, hemorrhage, reoperation, other surgical complications, Clavien-Dindo score [17], 30-day or in-hospital mortality, 90-day mortality and in-hospital stay were collected in the postoperative outcomes section. The item leakage included anastomotic or duodenal stump fistula and intra-abdominal abscess with a suspicion of an underlying fistula. Clavien-Dindo classification was also graded as no or minor postoperative morbidity (I and II) and major postoperative morbidity (III to V). Failure-to-rescue (FtR) was defined as the ratio between 90-day postoperative mortality and major postoperative morbidity.

### Outcomes

Primary outcomes were major postoperative morbidity, 30-day or in-hospital mortality, and 90-day mortality. Secondary outcome was the effect of age on short-term postoperative outcomes.

### Statistical analysis

Categorical variables were expressed as frequencies and percentages. Continuous variables were expressed as the median and the interquartile range. The chi-square test was used for the comparison of proportions and the Mann-Whitney test was used for the comparison of medians. The cumulative sum (CUSUM) chart was used to detect changes in postoperative mortality rates related to age. Funnel plot was used to detect variations of short-term postoperative outcomes among centers. Multivariable analysis was performed using a binary logistic regression. The statistical analysis was performed using the R version 4.0.3 (R Core Team, Vienna, Austria).

## Results

Initially, 726 patients that fulfilled inclusion criteria underwent surgery. Sixty-two (8.5%) patients did not follow surgical resection due to abdominal metastasis found during laparotomy, and additional 73 (10%) underwent only a palliative R2 resection. Ultimately, data from 591 (81.4%) patients in whom a resection with curative intent was accomplished could be analyzed (Fig. 1).

### Study population

Population characteristics are presented in Table 1. Five hundred and 48(92.7%) gastric carcinomas and 43 (7.3%) GEJ-SIII type carcinomas were analyzed. The antrum was the most frequent among gastric locations (49.4%) (Table 1). No center performed more than 15 gastrectomies annually and 10 of 17 hospitals participating in the study were very low-volume hospitals. Nevertheless, 366 (61.9%) patients were operated in hospitals performing 10 or more gastrectomies annually.

**Table 1:** Population, surgical, and tumor-related characteristics according to age

Patients	Total	< 75 years old	≥ 75 years old	***P*** value
	*n* = 591	*n* = 341 (57.7%)	*n* = 250 (42.3%)	
Sex				0.14
Men	363 (61.4%)	218 (63.9%)	145 (58%)	
Women	228 (38.6%)	123 (36.1%)	105 (42%)	
Location				0.001
GEJ-SIII type	43 (7.3%)	30 (8.8%)	13 (5.2%)	
Gastric	548 (92.7%)	311 (91.2%)	237 (94.8%)	
Fundus	35 (5.9%)	21 (6.2%)	14 (5.6%)	
Corpus	221 (37.4%)	145 (42.5%)	76 (30.4%)	
Antrum	292 (49.4%)	145 (42.5%)	147 (58.8%)	
ASA score				< 0.001
I	43 (7.3%)	38 (11.2%)	5 (2%)	
II	241 (40.9%)	168 (49.4%)	73 (29.3%)	
III	281 (47.7%)	129 (37.9%)	152 (61%)	
IV	24 (4.1%)	5 (1.5%)	19 (7.6.8%)	
Charlson	1 (0–2)	1 (0–2)	2 (1–3)	
Charlson categories				0.003
No or low (0–1)	332 (56.4%)	211 (62.2%)	121 (48.6%)	
Medium (2)	115 (19.6%)	61 (18%)	54 (21.7%)	
High (≥ 3)	141 (24%)	67 (19.8%)	74 (29.7%)	
Neoadjuvant therapy				< 0.001
No	424 (72.6%)	203 (60.2%)	221 (89.5%)	
Yes	160 (27.4%)	134 (39.8%)	26 (10.5%)	
Laparoscopic approach				0.92
No	531 (89.8%)	306 (89.7%)	225 (90%)	
Yes	60 (10.2%)	35 (10.3%)	25 (10%)	
Gastrectomy				< 0.001
Proximal	4 (0.7%)	3 (0.9%)	1 (0.4%)	
Distal subtotal	299 (50.9%)	134 (39.4%)	165 (66.5%)	
Total	285 (48.5%)	203 (59.7%)	82 (33.1%)	
Radicality				0.59
R0	559 (94.6%)	324 (95%)	235 (94%)	
R1	32 (5.4%)	17 (5%)	15 (6%)	
Lymphadenectomy				< 0.001
D0–D1	143 (24.3%)	56 (16.5%)	87 (34.9%)	
D1+ or more	445 (75.7%)	283 (83.5%)	162 (65.1%)	
Lymph nodes retrieved	17 (12–25)			
< 15 lymph nodes	232 (39.3%)	117 (34.4%)	115 (46%)	0.005
≥ 15 lymph nodes	358 (60.7%)	223 (65.6%)	135 (54%)	
Positive lymph nodes	329 (55.7%)			
pTNM classification				0.053
0	11 (1.9%)	9 (2.6%)	2 (0.8%)	
IA	72 (12.2%)	53 (15.5%)	19 (7.6%)	
IB	86 (14.6%)	46 (13.5%)	40 (16%)	
IIA	87 (14.7%)	46 (13.5%)	41 (16.4%)	
IIB	97 (16.4%)	58 (17%)	39 (15.6%)	
IIIA	73 (12.4%)	40 (11.7%)	33 (13.2%)	
IIIB	91 (15.4%)	46 (13.5%)	45 (18%)	
IIIC	74 (12.5%)	43 (12.6%)	31 (12.4%)	
Annual hospital volume				0.98
< 10	225 (38.1%)	130 (38.1%)	95 (38%)	
≥ 10	366 (61.9%)	211 (61.9%)	155 (62%)	

*GEJ-SIII* gastroesophageal junction Siewert type III, *ASA* American Society of Anesthesiologists classification

**Fig. 2:**
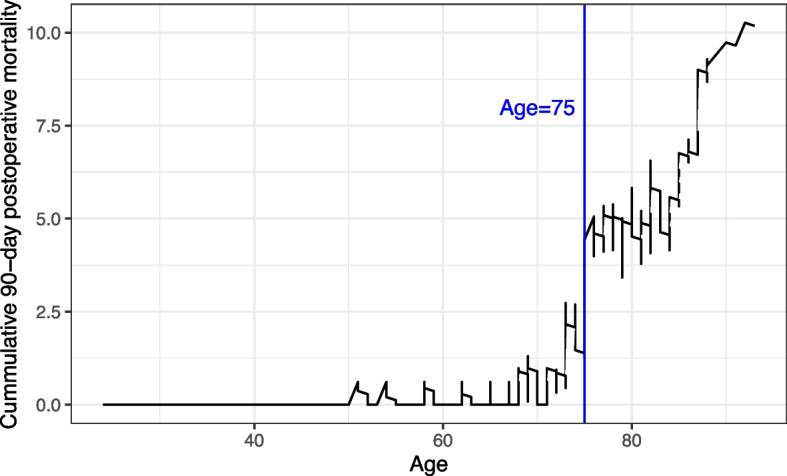
Cumulative mortality plot according to age

The median age of the whole study population was 71 (62–80), but significantly higher in patients with tumors located in the stomach compared to patients with GEJ-SIII tumors (72 vs 67 *p* = 0.019). Two age groups were established according to the cumulative sum plot depicted in Fig. 2. The risk of postoperative mortality increased steadily with age (*p* < 0.001), and after age 75, the observed probability exceeded the expected probability. The threshold established for 90-day postoperative mortality was 8.5% [18]. Patients aged 75 or older accounted for 42.3% of cases.

Men were more prevalent than women (61.4% vs 38.6%) among gastric and GEJ-SIII adenocarcinoma patients. Most patients were classified with ASA II (40.9%) or ASA III (47.7%) scores. No comorbidity or low Charlson score were more prevalent in this series (56.4%), but the Charlson score was higher in patients aged 75 years or older (*p* = 0.003) (Table 1).

### Surgical treatment and pathological features

A significantly higher proportion of patients younger than 75 years received neoadjuvant treatment (39.8%) in comparison with patients aged 75 or older (10.5%). Surgical and pathological data of the series are shown in Table 1. Overall, total and partial gastrectomy were performed in similar percentages, but total gastrectomy was more frequently performed in patients younger than 75 years (59.7% vs 33.1%). Only 60 (10.2%) patients were treated with a laparoscopic approach.

D1+ lymphadenectomy or a more extended lymph node resection was performed in 75.7% of the cases although it was carried out more frequently in patients younger than 75 years (83.5% vs 65.1%). The median number of retrieved lymph nodes was 17, and 15 or more lymph nodes were obtained in 60.7% of resections for pathological analysis. Positive lymph nodes were found in 55.7% of the lymphadenectomies, this percentage being higher in GEJ-SIII tumors (69%). Complete R0 resection was accomplished in 94.6% of the patients.

### Postoperative morbidity and mortality. Failure to rescue

Postoperative morbidity and mortality are summarized in Table 2. Major postoperative morbidity occurred in 154 (26.1%) patients, and it did not differ between very low-volume hospitals and centers with an annual caseload of 10 or more gastrectomies (26.7% and 25.7%).

**Table 2:** Postoperative complications and failure-to-rescue according to age

Patients	Total	< 75 years old	≥ 75 years old	***P*** value
	*n* = 591	*n =* 341 (57.7%)	*n* = 250 (42.3%)	
Clavien-Dindo classification				0.001
0–I	359 (60.8%)	224 (65.7%)	135 (54%)	
II	78 (13.2%)	39 (11.4%)	39 (15.6%)	
IIIa	37 (6.3%)	24 (7%)	13 (5.2%)	
IIIb	38 (6.4%)	24 (7%)	14 (5.6%)	
IVa	17 (2.9%)	11 (3.2%)	6 (2.4%)	
IVb	2 (0.3%)	1 (0.3%)	1 (0.4%)	
V	60 (10.2%)	18 (5.3%)	42 (16.8%)	
Morbidity grade				0.039
No or minor (I–II)	437 (73.9%)	263 (77.1%)	174 (69.6%)	
Major (III–V)	154 (26.1%)	78 (22.9%)	76 (30.4%)	
Pulmonary complications				< 0.001
No	502 (84.9%)	306 (89.7%)	196 (78.4%)	
Yes	89 (15.1%)	35 (10.3%)	54 (21.6%)	
Cardiac complications				0.004
No	548 (92.7%)	325 (95.3%)	223 (89.2%)	
Yes	43 (7.3%)	16 (4.7%)	27 (10.8%)	
Leakage				0.25
No	500 (84.7%)	294 (86.2%)	206 (82.7%)	
Yes	90 (15.3%)	47 (13.8%)	43 (17.3%)	
Hemorrhage				0.081
No	567 (96.1%)	324 (95%)	243 (97.6%)	
Yes	23 (3.9%)	17 (5%)	6 (2.4%)	
Reoperation				0.36
No	523 (88.6%)	306 (89.7%)	217 (87.1%)	
Yes	67 (11.4%)	35 10.3%)	32 (12.9%)	
Failure-to-rescue	60 (39%)	18 (23.1%)	42 (55.3%)	< 0.001
Mortality				< 0.001
30-day or in-hospital	52 (8.6%)	15 (4.4%)	37 (14.8%)	
90-day	60 (10.1%)	18 (5.3%)	42 (16.8%)	
In-hospital stay (days)	11 (8–18)			

Morbidity grade. *Minor* grade I and II of the Clavien-Dindo classification; *Major* grade III to V of the Clavien-Dindo classification

**Fig. 3:**
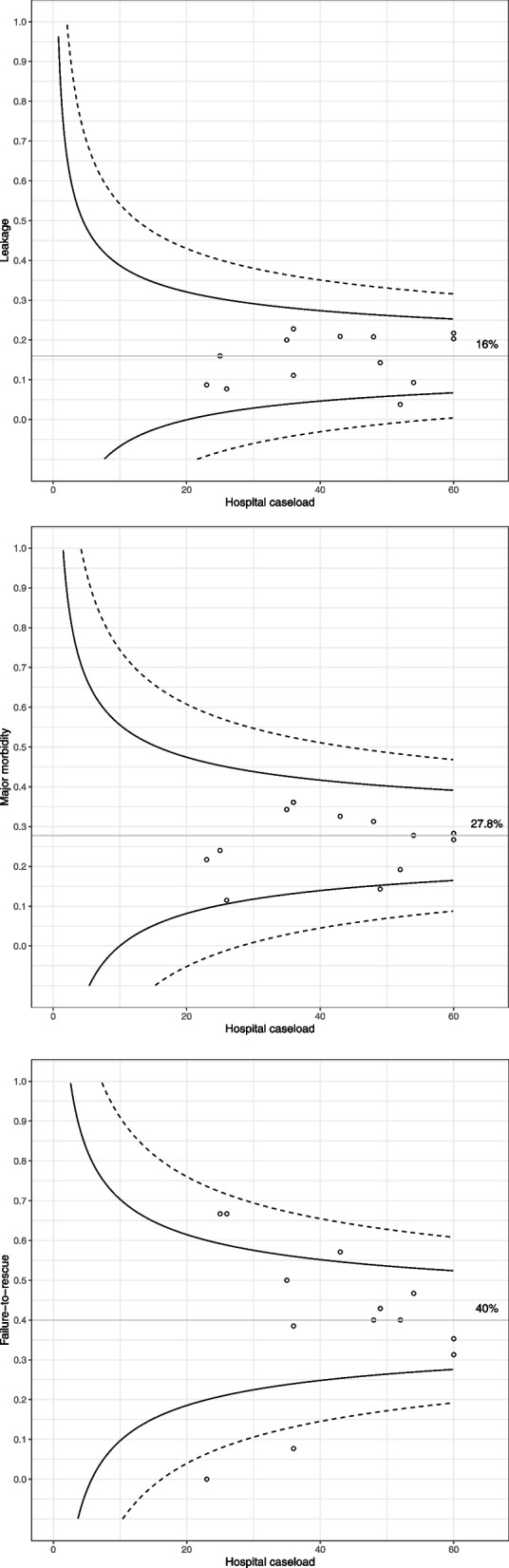
Funnel plots. Postoperative short-term outcomes by centers. Legend: **a** Leakage, **b** major morbidity, **c** failure-to-rescue

Patients aged 75 or older showed higher major postoperative morbidity in comparison with younger patients (30.4% vs 22.9% *p* = 0.039) (Table 2). Patients with GEJ-SIII tumors showed a trend towards a major postoperative morbidity rate compared to gastric tumors (37.2% vs 25.2% *p* = 0.084). Leakage occurred in 90 (15.3%) patients, somewhat higher but not statistically significant, in GEJ-SIII than in gastric tumors (23.8% and 14.6%). Sixty-seven (11.4%) patients were re-operated (GEJ-SIII 9.5% and gastric 11.5%) being in 43 (64.2%) of them associated with leakage. FtR occurred in 60 (39%) patients and it was higher in patients aged 75 or older in comparison with younger patients (55.3% vs 23.1%, *p* < 0.001). Variations among hospitals regarding leakage, major postoperative morbidity and failure-to-rescue rates are shown in Fig. 3a-c.

The univariable analysis showed that age 75 years or older (*p* = 0.039), ASA score (*p* = 0.034), neoadjuvant chemotherapy (*p* = 0.036), laparoscopic approach (*p* = 0.022), and type of gastrectomy (*p* = 0.015) were associated with major postoperative morbidity (Table 3). According to the results of the univariable analysis the following variables entered the multivariable analysis: age, sex, location, ASA score, neoadjuvant chemotherapy, laparoscopic approach, type of gastrectomy and type of lymphadenectomy. Age 75 years or older (OR 1.62, 95% CI 1.05–2.49), laparoscopic approach (OR 2.32, 95% CI 1.29–4.17), and total gastrectomy (OR 1.86, 95% CI 1.2–2.86), emerged as independent prognostic factors for major postoperative morbidity (Table 4).

**Table 3:** Major postoperative morbidity. Univariable analysis

	No	Yes	***p value***
	*n* = 437 (73.9%)	*n* = 154 (26.1%)	
Age (years)	70 (61–79)	74 (65–81)	0.012
Age categories			0.039
< 75	263 (77.1%)	78 (22.9%)	
≥ 75	174 (69.6%)	76 (30.4%)	
Sex			0.07
Men	259 (71.3%)	104 (28.7%)	
Women	178 (78.1%)	50 (21.9%)	
Location			0.084
GEJ-SIII type	27 (62.8%)	16 (37.2%)	
Gastric	410 (74.8%)	138 (25.2%)	
ASA score			0.034
I	33 (76.7%)	10 (23.3%)	
II	189 (78.4)	52 (21.6%)	
III	200 (71.2%)	81 (28.8%)	
IV	13 (54.2%)	11 (45.8%)	
Charlson	1 (0–2)	1 (0–2)	0.52
Charlson categories			0.896
No or low (0–1)	247 (74.4%)	85 (25.6%)	
Medium (2)	83 (72.2%)	32 (27.8%)	
High (> 2)	104 (73.8%)	37 (26.2%)	
Neoadjuvant therapy			0.036
No	303 (71.5%)	121 (28.5%)	
Yes	128 (80%)	32 (20%)	
Laparoscopic approach			0.022
No	400 (75.3%)	131 (24.7%)	
Yes	37 (61.7%)	23 (38.3%)	
Gastrectomy			0.015
Proximal	1 (25%)	3 (75%)	
Distal subtotal	232 (77.6%)	67 (22.4%)	
Total	202 (70.9%)	83 (29.1%)	
Resection			0.784
R0	414 (74.1%)	145 (25.9%)	
R1	23 (71.9%)	9 (28.1%)	
Lymphadenectomy			0.085
D0–D1	99 (69.2%)	44 (30.8%)	
D1+ or more	336 (75.5%)	109 (24.5%)	
pTNM classification			0.739
0	10 (90.9%)	1 (9.1%)	
IA	51 (70.8%)	21 (29.2%)	
IB	65 (75.6%)	21 (24.4%)	
IIA	69 (79.3%)	18 (20.7%)	
IIB	69 (71.1%)	28 (28.9%)	
IIIA	54 (74%)	19 (26%)	
IIIB	67 (73.6%)	24 (26.4%)	
IIIC	52 (70.3%)	22 (29.7%)	
Annual hospital volume			0.791
< 10	165 (73.3%)	60 (26.7%)	
≥ 10	272 (74.3%)	94 (25.7%)	

*GEJ-SIII type* gastroesophageal junction Siewert type III, *ASA* American Society of Anesthesiologists classification

**Table 4:** Postoperative outcomes. Multivariable logistic regression analysis

	OR (95% CI)	***p value***
**Major postoperative morbidity**		
Age (years)		0.029
< 75 (ref.)		
≥ 75	1.62 (1.05–2.49)	
Sex		0.183
Men (ref.)		
Women	0.76 (0.51–1.14)	
Location		0.293
GEJ-SIII type (ref.)		
Gastric	0.67 (0.32–1.4)	
ASA score		0.24
I–II (ref.)		
III–IV	1.28 (0.85–1.93)	
Neoadjuvant chemotherapy		0.065
No (ref.)		
Yes	0.62 (0.38–1.03)	
Laparoscopic approach		0.005
No (ref.)		
Yes	2.32 (1.29–4.17)	
Gastrectomy		0.005
Distal subtotal (ref.)		
Total	1.86 (1.2–2.86)	
Lymphadenectomy		0.445
D0–D1 (ref.)		
D1+ or more	0.84 (0.53–1.32)	
**90-day postoperative mortality**		
Age (years)		0.027
< 75 (ref.)		
≥ 75	2.42 (1.15–5.29)	
ASA score		0.093
I–II (ref.)		
III–IV	1.98 (0.89–4.39)	
Laparoscopic approach		0.495
No (ref.)		
Yes	1.43 (0.51–3.97)	
Gastrectomy		0.769
Distal subtotal (ref.)		
Total	0.89 (0.42–1.88)	
Pulmonay complications		< 0.001
No (ref.)		
Yes	8.54 (4.17–17.47)	
Cardiac complications		< 0.001
No (ref.)		
Yes	5.14 (2.09–12.61)	
Leakage		0.003
No (ref.)		
Yes	3.56 (1.56–8.12)	
Hemorrhage		0.013
No (ref.)		
Yes	5.1 (1.4–18.48)	
Reoperation		0.136
No (ref.)		
Yes	1.99 (0.81–4.9)	

*GEJ-SIII type* gastroesophageal junction Siewert type III, *ASA* American Society of Anesthesiologists classification

Overall 30-day or in-hospital mortality, and 90-day postoperative mortality rates were 8.6% and 10.1% respectively. Postoperative mortality did not differ either between very low-volume hospitals and centers with an annual caseload of 10 or more gastrectomies (9.3% and 10.7%). The 90-day postoperative mortality did not differ depending on GEJ-SIII or gastric location but it was significantly higher in patients aged 75 years or older in comparison with younger patients (16.8% vs 5,3% *p* < 0.001) (Table 2). Pulmonary complications and cardiac complications occurred in 63% and 38% of patients who subsequently died. Multivariable analyses that included the variables age, ASA score, laparoscopic approach, type of gastrectomy, pulmonary complications, cardiac complications, leakage, hemorrhage and reoperation identified age 75 years or older (OR 2.42, 95%CI 1.15–5.29), pulmonary complications (OR 8.54, 95% CI 4.17–17.47), cardiac complications (OR 5.14, 95% CI 2.09–12.61), leakage (OR 3.56, 95% CI 1.56–8.12), and hemorrhage (OR 5.1, 95% CI 1.4–18.48) as independent risk factors of 90-day postoperative mortality (Table 4).

## Discussion

The implementation of the RECEG-CV allowed to analyze overall short-term postoperative outcomes in gastric adenocarcinoma patients treated in 17 out of the 24 hospitals of the Public Health Service of the Comunitat Valenciana and to identify variations among hospitals as well. In this first assessment of the data registered during a 4-year period, 30-day and in-hospital mortality, and 90-day mortality rates were 8.6% and 10.1% respectively. The fact that all patients included in our registry were treated in low-volume centers might partly explain the higher mortality rate compared to that reported in other clinical registries that include patients treated in high-volume centers. In a study gathering data from 5 European countries the 30-day and in-hospital mortality after gastrectomy ranged from 2.2 to 7.2% [6]. However, 30-day and in-hospital mortality rates may underestimate the true postoperative mortality, and 90-day mortality is deemed more accurate in assessing surgery-related mortality [19, 20]. Thus, a recent report from the nationwide Swedish Gastric Cancer Surgery Study (SWEGASS) showed an outstanding 30-day mortality rate of 2.9% but 90-day mortality worsened remarkably to 7.1% [21]. This significant difference between 30- and 90-day postoperative mortality (4.6 and 8.6%) was also shown in a nationwide French study [18]. Altogether, data from European clinical registries show that postoperative mortality of gastric carcinoma patients remains high. Different variables related to patient and center characteristics have been associated with postoperative mortality.

The effect of age on postoperative mortality is controversial because different cutoffs have been used in the different studies. In our study, the CUSUM plot detected a significant increase of postoperative mortality from age of 75 on, and this age cutoff point emerged also as an independent factor associated with postoperative mortality in the multivariable analysis. In our cohort this age group constituted 42% of the patients, which might also explain to a certain extent the high postoperative mortality observed in our series. In another population-based study, 90-day postoperative mortality of gastric cancer patients aged 75 years and older was 16% [19], similar to the one observed in our cohort. A recent report of the Dutch Upper Gastrointestinal Cancer Audit (DUCA) nationwide registry showed that age 70 and older was on the verge of significant association with postoperative 30-day or in-hospital mortality (OR 1.56; CI 95% 0.99 to 2.46) [21]. A progressive increase in postoperative mortality above 70 years of age was also observed within the CUSUM plot in our cohort, but significance was only reached at a cutoff point of 75 years. In addition, older patients suffered higher comorbidity and often underwent less extended surgical procedures which are potential confounding factors for assessment the effect of age on postoperative mortality. However, ASA score and total gastrectomy were not associated with postoperative mortality in the multivariable analysis. Comorbidity has been associated to an increase of postoperative mortality in some studies [11, 22], but ASA score and Charlson index may depend on interobserver assignment variability. Our results suggest that other factors associated with age such as frailty might also explain the sharp increase of postoperative mortality observed in patients aged 75 or older. Unfortunately, a frailty score was not included as a variable in our registry. Probably, identifying frail older patients during the preoperative study would be helpful for assessing more carefully the risk of postoperative morbidity and mortality [23, 24].

Neoadjuvant chemotherapy was less frequently administered than reported by the DUCA registry during the same period [21], and it was seldom administered to patients aged 75 years and older. Also, patients following neoadjuvant chemotherapy showed a not statistically significant trend to a lower major postoperative morbidity rate, in line with that described in the previous study [21], suggesting that patients in better preoperative condition received more often chemotherapy. Pulmonary and cardiac complications, leakage, and hemorrhage were independent risk factors of postoperative mortality, as reported previously in other studies [25–27]. Anastomotic leakage rates from 4.9% to 9.8% have been reported in several European registries [25–27], but overall leakage rates may have been underestimated in them, because intra-abdominal abscess, or peritonitis were recorded separately. In our analysis, leakage rate was not higher in patients aged 75 or older or in hospitals with an annual caseload below 10 either, although marked variations were observed among hospitals. Quality standards in postoperative outcomes have been defined for some digestive neoplasias but not yet for gastric cancer surgery [28, 29]. Outstanding postoperative clinical outcomes after gastrectomy for gastric cancer have been reported by the Japanese nationwide registry [30] but this cannot be presumably taken as a benchmark in Western countries. Recently, the European Gastric Cancer Association created the Gastrectomy Complications Consensus Group, with 27 participating gastric cancer referral centers. It developed a list of postoperative complications and reported short-term postoperative outcomes that might be helpful as a benchmark for gastric cancer surgery [27]. However, it should be taken into account that only selected centers participated in this project.

Patients with gastric carcinoma have a remarkable risk of death once postoperative major morbidity occurs and therefore, FtR has emerged as an important postoperative outcome in assessing the quality of surgical treatment. In our cohort, while major postoperative morbidity was only somewhat higher in patients aged 75 years or older (30% *versus* 23%), the FtR recorded was more than double for that group compared to younger patients (55% *versus* 23%). In addition, the FtR outcome detected remarkable differences among hospitals that might provide relevant information about the capacity to solve major postoperative complications in each center. These findings suggest that both age and center-related characteristics may have influence on this outcome. However, FtR should be analyzed with caution when the sample size of patients with major postoperative morbidity in a given hospital is relatively small. In a report from the DUCA registry where only 30-day and in-hospital mortality was considered, the FtR was initially 38% but this rate halved at the end of the 4-year study period [25]. Interestingly, a process of centralization was launched in The Netherlands during this period. Similarly, a report from the German nationwide registry showed a reduction of FtR associated with annual caseload [26].

Centralization policies for gastric cancer surgery have been launched in several European countries, and a minimum annual caseload of 20 gastrectomies has been proposed to achieve optimal short-term postoperative outcomes. We could not analyze the beneficial effect of annual hospital volume on short-term postoperative outcomes because only 7 centers performed 10 or more gastrectomies annually and none performed more than 15 during the study period. Thus, it might be proposed that poor rates in some short-term postoperative outcomes are related to this fact. In a report showing the short-term results after the implementation of the DUCA registry a decrease of 30-day mortality (7.1 to 4.3%) was observed at the end of the 8-year period [10]. During this period a progressive decrease of hospitals with an annual caseload below 20 gastrectomies occurred due to the centralization policy initiated in The Netherlands. Nevertheless, both, the implementation of the registry and the progressive centralization policy might have had an impact on the improvement of postoperative outcomes. In a study comparing short-term postoperative outcomes between the DUCA registry and the Swedish National Register for Oesophageal and Gastric Cancer (NREV) [12] postoperative morbidity and 30-day or in-hospital mortality was better in Sweden despite only 18% of the patients of the Swedish registry being surgically treated in hospitals performing over 20 gastrectomies per year. Nevertheless, a process of centralization of gastric cancer was formally recommended in Sweden afterwards [31]. Although centralization of gastric cancer patients is still a matter of debate, some nationwide population studies carried out in countries where centralization is not implemented suggest a beneficial effect on short-term postoperative outcomes in hospitals with a high annual volume of gastric resections [18, 26]. Moreover, minimally invasive surgical approach, increasingly introduced for the treatment gastric cancer, has a learning curve of around 40 procedures for laparoscopic distal gastrectomy and even more for laparoscopic total gastrectomy [32, 33]. In our cohort, all centers that performed laparoscopic gastrectomies were still within the learning curve which may explain that this procedure was associated with a higher postoperative major morbidity. It should also be considered that the progressive decrease of gastric carcinoma incidence in Western countries may add difficulties in completing the learning curve in low-volume centers.

Our study has some limitations. None of the hospitals performed more than 15 gastrectomies per year, and therefore the possible effect of centralization gastric cancer treatment in high-volume centers could not be analyzed. Also, participation in the RECEG-CV was optional and 7 of the 24 hospitals of the public network did not include patients in the RECEG-CV, but these 7 centers perform a low or very low volume of gastric resections annually. Finally, we did not perform a random sample verification. However, two investigators examined data carefully to detect those that were implausible or mistaken.

## Conclusions

Our analysis shows that centralization of gastric cancer treatment in centers with higher annual caseload should be considered to improve the short-term postoperative outcomes in low-volume centers. Also, patients aged 75 or older had a significantly increased risk of major postoperative morbidity and mortality with a remarkably higher FtR, indicating that these patients should be more carefully studied before undergoing oncological gastrectomy. Finally, the implementation of the RECEG-CV was able to evaluate as a whole the studied short-term postoperative outcomes, also detecting noteworthy variations between the participating hospitals. Therefore, data supply to this registry should be required henceforth for all centers performing gastric cancer surgery in our autonomous region.

## Data Availability

The datasets generated are not publicly available because they are in the Esophagogastric Cancer Registry of the Comunitat Valenciana (RECEG-CV). The RECEG-CV is a population-based surgical online registry fostered by the Valencian Surgical Society and the Epidemiological Studies and Health Statistics Service of the Comunitat Valenciana (Spain). The RECEG-CV is included into the NEOS platform that belongs to the Cancer Information System of the Comunitat Valenciana which guarantees the compliance with the data protection law. The analyzed data of the current study could be available from the corresponding author on reasonable request.

## Abbreviations

GEJ: Gastroesophageal junction
GEJ-SIII: GEJ Siewert III type
RECEG-CV: Esophagogastric Cancer Registry of the Comunitat Valenciana
SEEGCR: Spanish EURECCA Esophagogastric Cancer Registry
FtR: Failure to rescue
CUSUM: Cumulative sum chart
ASA: American Society of Anesthesiologists
DUCA: Dutch Upper Gastrointestinal Cancer Audit
